# The integration of local stilt house architecture in natural disaster mitigation education in South Sumatra, Indonesia

**DOI:** 10.4102/jamba.v18i1.1987

**Published:** 2026-01-28

**Authors:** Yusni Arni, Elsi A. Fitri, Chika Rahayu, Darsin Darsin

**Affiliations:** 1 Master of Elementary Education, Faculty of Teacher Training and Education, Universitas PGRI Palembang, Palembang, Indonesia; 2 Department of Science Education Study Programme, Faculty of Teacher Training and Education, Universitas PGRI Palembang, Palembang, Indonesia; 3 Magister Mathematics Education, Faculty of Teacher Training and Education, Lampung University, Lampung, Indonesia; 4 Information Systems Study Program, Faculty of Computer Science, Institute of Business Technology and Languages Dian Cipta Cendikia, Lampung, Indonesia

**Keywords:** ethnoscience, disaster mitigation, disaster education, stilt houses, South Sumatra

## Abstract

The areas of Sumatra are multidisaster-prone zones, especially to floods and earthquakes. However, these regions also preserve local wisdom, particularly through stilt house architecture, which serves as a community-based adaptive strategy. This study aims to explore the function of local stilt houses as a form of disaster mitigation and assess the younger generation’s knowledge regarding the functional and philosophical aspects of stilt houses, as well as their potential for integration into disaster education. A mixed-methods approach was employed, involving in-depth observations and interviews with community figures, along with the distribution of questionnaires to 158 students across South Sumatra. The study findings show that traditional stilt houses not only withstand flood and earthquake risks but also reflect socio-cultural, spiritual and environmental sustainability values. Their structural designs, use of local materials, house orientation and tiered-room concept serve as clear representations of ethnoscience in local architectural practice. The questionnaire results indicate that the majority of the younger generation possesses moderate to high understanding of the social and functional aspects of stilt houses but lacks sufficient knowledge of their symbolic and technical dimensions. These findings highlight the importance of integrating local knowledge into disaster education curricula to strengthen community resilience from an early age and preserve proven cultural adaptations to disaster risk.

**Contribution:**

This article contributes to disaster risk reduction efforts by integrating the local wisdom of stilt house architecture into science education. It promotes a culturally grounded approach to flood and earthquake mitigation through the use of ethnoscience in educational contexts.

## Introduction

Indonesia is one of the countries with the highest disaster potential, where 5400 natural disasters have occurred in the period 2020–2023 (Dibi.bnpb 2024). This is due to Indonesia’s location along the Pacific Ring of Fire, at the convergence of the Indo-Australian, Eurasian, and Pacific tectonic plates, which results in high tectonic activity with plate movements of approximately 4–6 cm per year, particularly in coastal areas directly facing the open sea (Akbar et al. 2020; Alimsuardi, Suprayogi & Amarrohman 2019; Ayunda et al. 2020; Hargono et al. 2023; Irwanto 2015; Kartini, Widodo & Winarno 2021; Kastolani & Mainaki 2018; Marlina, Ruslanjari & Hakim 2024). This geographical condition is what often causes natural disasters such as earthquakes, landslides, floods, tsunamis and others. The coast of Sumatra is a multidisaster-prone area, especially earthquakes and floods. Geologically, this region lies along the subduction zone of the Indo-Australian and Eurasian plates, which are the main triggers for high-intensity earthquakes. Over the past two decades, there have been more than 150 significant earthquakes and more than 200 major floods that hit the coastal region of Sumatra (Badan Nasional Penanggulangan Bencana 2024).

Some of the coastal areas of Sumatra that are included in the vulnerable zone include Bengkulu, which has historically experienced major earthquakes due to active subduction zones off its coast; the coast of West Sumatra, which is one of the areas most at risk of tsunami and was affected by a major earthquake in 2009; Lampung, which is close to the Sunda Strait and prone to volcanic activity and underwater earthquakes, and Aceh, which experienced a megathrust earthquake and tsunami tragedy in 2004 that hit the coast of Aceh and spread to 12 neighbouring countries (Agussaini 2022; Gadeng 2018; Ghobarah, Saatcioglu & Nistor 2006; Havwina, Maryani & Nandi 2017; Syamsidik 2021). In addition, flash floods in Agam and West Pasaman in 2022, as well as cold lava floods from Mount Marapi in 2024, show that coastal areas are not only at risk of earthquakes but also other hydrometeorological and geological disasters that reinforce each other’s impacts (Narny 2025; Wiryanto 2025). The impact of the earthquake and tsunami was very devastating and became one of the largest cases in world history, especially in the East and Southeast Asian regions. This disaster took the lives of more than 300 000 people and caused enormous damage to infrastructure (Ghobarah et al. 2006). The vulnerability of disasters in the region shows that mitigation efforts can not only rely on modern structural approaches but also require adaptation strategies rooted in local social and ecological contexts.

In this context, local wisdom needs to be operationally affirmed as knowledge, values and practices that develop from generation to generation through community interaction with their environment, which has proven to be effective in maintaining the sustainability of life and reducing disaster risk (Basri 2021; Triastari 2021). Local wisdom not only is in the form of cultural traditions but also includes technical adaptations, ecological practices and social rules that empirically help communities survive environmental dynamics (Anwar 2021; Utami et al. 2025).

The latest disaster to shake the coastal region is the massive flood that occurred in May 2025 in the eastern coastal region of South Sumatra, including Banyuasin and Ogan Ilir Regencies. Extreme rainfall that persisted for 5 days resulted in the overflow of the Musi River and its tributaries. On the other hand, the topographic character and poor land use also make the Sumatran very vulnerable to annual floods and tidal floods. Large rivers such as the Musi River and Batang Arau are the main channels that easily overflow during the rainy season or when the sea level experiences high tide. Data from the South Sumatra BPBD recorded that more than 12 000 houses were submerged, and also educational institutions, health facilities and main roads. In addition to physical damage, these disasters also have health impacts, such as increasing cases of acute respiratory infections (ARI) and skin diseases due to polluted environments and inappropriate evacuations (Handika 2023). This case reinforces the reality that floods have become an annual disaster in South Sumatra.

Flood disasters in coastal areas not only occur due to extreme rainfall factors but are also exacerbated by environmental degradation and watershed damage. In Palembang, for example, a study (Putra et al. 2023) indicates that some sub-watersheds are experiencing high pressure due to sedimentation and poor drainage systems, which lead to routine flooding in densely populated areas. Geographically, the South Sumatra region has an alluvial soil structure and extensive river network, making it particularly vulnerable to annual flooding (Handika 2023; Sandika 2025). On the other hand, although the intensity of earthquakes in this region is not as active as West Sumatra or Bengkulu, the Palembang area and its surroundings remain within the radius of the influence of active faults such as the Semangko Fault. According to (Pratama 2025), medium-scale earthquakes (M 5.0–6.0) that occur in this transition zone can have an impact on low-level building structures and are not shock resistant, especially in densely populated areas with makeshift construction. Therefore, the people of South Sumatra are in a position to be structurally vulnerable to a combination of natural disaster threats.

One of the tangible forms of ethnoscience in the Sumatra region is stilt house architecture (Fitri & Arni 2024). The architecture of stilt houses in South Sumatra actually developed strongly in communities living on the banks of the river, especially along the Musi River, one of the largest rivers in Sumatra. The pattern of community settlements that are centred on the mainland but oriented towards the river makes the stilt house a logical ecological adaptation to the dynamics of water levels, seasonal flooding and alluvial soil conditions. Traditional stilt houses built by the community not only reflect cultural values but also contain scientific principles in disaster mitigation. The stilt house is designed to minimise the impact of floods and earthquakes. In addition, the space beneath the stilt house functions as a buffer zone that reduces water runoff during flooding and acts as a flexible structural system, allowing the wooden framework to absorb and dissipate seismic energy during earthquakes.

Unfortunately, this local knowledge is beginning to be forgotten and is no longer of interest to the younger generation, so it has the potential to be forgotten and even lost. Based on direct observation of the community, especially the younger generation, it is known that 78% of respondents know that the stilt house is only a traditional architectural heritage, while only 22% understand that the design is related to flood mitigation and structural stability. The lack of understanding of the younger generation on the functional aspects of stilt houses shows a significant knowledge gap and emphasises the need to integrate local wisdom in context-based disaster mitigation education. This condition is a serious challenge, especially in preserving local wisdom while minimising the impact of natural disasters.

So that one of the efforts that can be made to answer these problems can be done by integrating natural disaster mitigation through education. Mitigation education is an important step in preparing a culture of disaster preparedness, including for school-age and school-age children as *agents of change* to prepare a disaster-resilient society (Weiland 2021). Promoting mitigation education is an effort to empower the community as part of the culture of disaster mitigation preparedness. This also supports the government’s programme, namely the Disaster Preparedness School Programme (SSB), which is one of the strategic efforts to strengthen school capacity in dealing with natural disaster risks in a systematic and sustainable manner.

Based on these problems, the purpose of this research is to examine in-depth the local wisdom of stilt house architecture as a strategy to reduce the risk of floods and earthquakes in South Sumatra and to find out the level of knowledge and perception of the younger generation on the mitigative function and cultural value of stilt houses, in order to formulate integration recommendations in disaster education.

## Research methods and design

This study employs a mixed-methods approach, combining both qualitative and quantitative methods to gain a comprehensive understanding of the local wisdom of Sumatran society regarding stilt house architecture as a disaster mitigation strategy against floods and earthquakes. It also examines the extent to which this concept is understood and interpreted in educational contexts by the younger generation, particularly students.

The qualitative approach focuses on deeply exploring cultural values, socio-economic functions and the historical and adaptive significance of stilt houses in the daily lives of local communities. Integration is carried out using *a sequential explanatory strategy*. Data collection was conducted through direct observations of stilt houses located in flood- and earthquake-prone areas along the Musi River, as well as in-depth interviews with traditional elders, community leaders, village officials and long-time residents of these stilt houses. The qualitative sample was determined using *purposive sampling* with the criteria of living in a stilt house for at least 15 years, having knowledge of customs, local history or construction practices and being willing to be interviewed. These individuals are traditionally recognised as key community figures, including the *Pasirah* or *Depati* [customary leaders], *Kyai* [religious leaders] and Traditional Elders (guardians of local values and norms) (Amri 2022). The number of informants consisted of 18 resource persons with four traditional elders (2 men and 2 women), three *Pasirah/Depati*, three *Kyai* [religious shops] and eight senior residents of stilt houses. Gender distribution: 10 males and eight females, considered to maintain diversity of socio-cultural experiences. Additional information was gathered from document analysis, including historical archives, customary records and local policies related to stilt house preservation and disaster mitigation efforts. Interviews were conducted in person using both Indonesian and local Sumatran dialects, either in group settings or individual sessions, to understand local terminologies and context-specific values. Qualitative data were analysed thematically to identify meaningful patterns that reflect the interconnection between culture, the architectural structure of stilt houses and community adaptation strategies to flood and earthquake disasters.

Meanwhile, the quantitative approach was used to measure students’ level of knowledge and perception regarding the design of stilt houses as a form of local wisdom relevant to disaster education. Sample selection was done using *stratified random sampling* based on faculty and study programme. Quantitative data were collected through the distribution of questionnaires to 158 students from various study programmes, particularly those in Science Education and Elementary School Teacher Education (PGSD) across the provinces of South Sumatra and Lampung. The questionnaire was developed based on the findings from the qualitative phase and a review of relevant literature. It included indicators related to knowledge of natural disasters, the structural design of stilt houses, cultural and social values, the economic function of stilt houses and the relevance of these concepts within educational contexts.

The research instrument consisted of 15 items developed using a four-point Likert scale (Likert 1932). The knowledge levels were categorised into four groups: high, sufficient, minimal and no knowledge. The validity of the instrument was tested using Pearson correlation, by comparing the correlation of each item with the total score. The validity test was conducted on a sample of 30 students. Based on the analysis, 14 items were found to be valid, while 1 item was considered invalid and subsequently removed.

The remaining 14 valid items were then subjected to a reliability test using Cronbach’s alpha. The results showed a reliability coefficient of α = 0.870, which is categorised as high reliability. According to Sugiyono (2019), an instrument is considered to have good reliability if the Cronbach’s alpha value exceeds 0.70 (Sugiyono 2019). In addition, the instrument was also tested for validity content by *three* experts (disaster mitigation and science lecturers) who assessed the suitability of the indicators with the theoretical construct of local wisdom and mitigation. This result indicates that the instrument used has strong internal consistency in measuring the intended construct.

The data integration process is carried out through a triangulation strategy in which qualitative findings are used to explain quantitative patterns, while quantitative results help confirm the generalising power of qualitative findings. Thus, the two data do not stand alone but complement each other: qualitative data explain the ‘why’ and ‘how’ local wisdom works as disaster mitigation, while quantitative data show the extent to which such knowledge is understood by the mud generation. All data, both primary and secondary, are interpreted into narratives that affirm the relationship between culture, disaster risk and its implications for disaster risk reduction (DRR) education.

This study began with an introductory phase consisting of a literature review and oral history exploration. This phase aimed to formulate a framework for integrating local wisdom, specifically stilt house architecture into disaster education, as an effort to preserve cultural heritage and enhance disaster preparedness among the younger generation. Next, data collection was conducted and refined to obtain valid datasets, consisting of both primary and secondary sources. Primary data were obtained from the experiences of informants or eyewitnesses, supported by their opinions and written materials from the relevant time period, serving as direct sources of knowledge. Secondary data included the results of previous research, scholarly articles, books and other documented materials used as additional references. The qualitative data collected were selectively integrated with the quantitative data obtained from the questionnaire results to form a comprehensive analysis.

The analysis and interpretation of the collected data were carried out to generate meaningful insights. Qualitative data were analysed thematically, taking into account religious and customary backgrounds, along with other influencing factors. Meanwhile, quantitative data were analysed using descriptive statistics.

**FIGURE 1:**
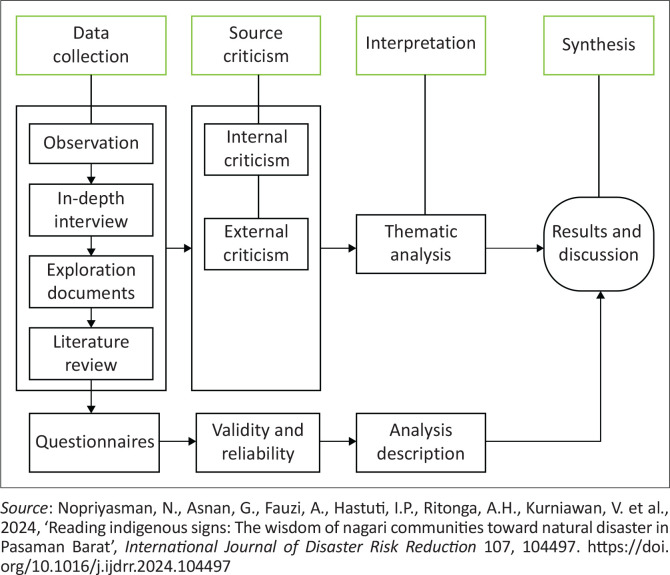
Research procedures.

These meaningful findings were then synthesised into a coherent narrative that highlights their significance in reducing the risks of floods and earthquakes in Sumatra, particularly in South Sumatra. The historical findings of this study can serve as valuable lessons for society, illustrating how communities can take wise and informed actions in critical situations. This cultural knowledge is reflected in the management and application of local wisdom, especially in relation to the interconnection between society, disaster contexts and education. The overall stages of this process are illustrated in Figure 1.

### Ethical considerations

Ethical clearance to conduct this study was obtained from the Research Ethics Committee of PGRI University of Palembang (No. 454/F.30/LPPKM/UNIV.PGRI/2025).

## Results

### Local community knowledge related to natural disasters

In response to the two major types of disasters, earthquakes and floods, the communities of Sumatra have developed local architectural adaptations in the form of traditional stilt houses (*rumah panggung*). These houses represent both adaptive and mitigative strategies grounded in local wisdom. Traditional stilt houses are generally constructed using flexible wooden structures and are elevated on poles, allowing floodwaters to flow beneath the floor of the house. In the context of earthquakes, the lightweight and elastic design of stilt houses provides greater resilience in absorbing seismic vibrations compared to rigid concrete buildings (Anwar 2025; Basri 2021). The elevation of stilt houses typically ranges from 1 m to 2 m, enabling them to remain safe even when floodwaters rise to knee or waist level in adults.

Interviews with community figures and analysis of local documents reveal that the construction of stilt houses is not merely a technical decision but also deeply embedded with cultural and spiritual significance. Certain local myths, such as folktales about ‘swaying land’ and ‘red water’ in West Sumatra, are interpreted as symbolic early warnings of impending disasters. This traditional wisdom reinforces the understanding that stilt houses are part of a local knowledge system that has been empirically proven to reduce losses during disasters (Slamet 2025). These houses are also regarded as symbols of preparedness, as their construction requires careful mapping of safe locations and the appropriate selection of building materials.

### Structural characteristics of stilt houses

The architecture of stilt houses along the South Sumatra is specifically designed to minimise hydrological impacts by elevating the main floor approximately 1 m – 2 m above the ground level. This height is not just an aesthetic or status symbol but is based on hereditary observations of the overflow pattern of river water. This happens because it can reduce exposure to flooding as water can flow through the basement of the house, reducing the lateral pressure of the water, thus preventing structural damage and can facilitate underground ventilation to accelerate drying after a flood. This height has been proven effective in withstanding the annual floodwaters of the Musi River, which typically reach between 0.6 m and 1.0 m, thereby significantly reducing damage to household contents. A study by Arifin, Wunas and Mushar (2022) on coastal settlements in Sulawesi found that every additional 50 cm in pole height can reduce economic losses to stilt houses by up to 18% (Arifin et al. 2022). This finding aligns with conditions in South Sumatra, where the open space beneath the house functions as a buffer zone for water flow, sediment and even tidal currents. The width of this underfloor gully also ensures proper air circulation, facilitates faster drying after floods and helps prevent wood corrosion. These features provide clear ecological advantages compared to unsustainable concrete structures built directly on the ground. As noted by Basri (2021), the presence of open space beneath the floor not only enables economic activities to continue during floods but also serves as a rapid evacuation point when disasters strike (Basri 2021).

Traditional stilt house construction in the areas of Sumatra utilises various types of wood as the primary building materials, with each type selected based on its specific function and durability. Exciting wood (a local term for durable hardwood) is commonly used for the main framework due to its strength in supporting the overall structure of the house. For the foundation, communities typically use *unglen* wood, which is known for its high resistance to humidity and minimal susceptibility to weathering, even when frequently exposed to standing water. Meanwhile, *tembesu* wood is often used for constructing floors, walls, windows and doors, owing to its strength, durability and ability to withstand tropical weather conditions. In terms of earthquake resilience, *the combination of exciting and tembesu woods*, joined using mortise and tenon joints, results in a lightweight and flexible structure. This design places the building’s natural period below the peak response spectrum of moderate earthquakes (Mw 5–6). This principle aligns with the findings of Hildayanti and Wasilah (2021), which showed that post-disaster stilt houses in Lombok experienced 35% less structural damage compared to conventional masonry houses (Hildayanti & Wasilah 2021). Moreover, *unglen* wood used in the foundation exhibits semi-ductile behaviour, enabling it to distribute seismic vibrations into the ground without forming brittle cracks, while also withstanding long-term water exposure. Unglen wood is able to distribute earthquake energy to the ground gradually, where the pole is directly inserted and provides lateral friction that adds stability to soft soil.

The selection of wood materials reflects the community’s wise and sustainable use of natural resources. In addition to their high functional value, these wood materials support environmentally friendly principles, as they can last for extended periods when properly treated. During construction, the joints between structural components are typically connected using wooden nails. The main structural posts are installed by being driven into the ground using wooden hammers, which helps prevent damage to both the tools and the wood materials. This method, hammering wooden nails with wooden tools rather than metal, preserves the integrity of the wood fibres, thereby enhancing the strength and cyclic durability of the joints.

**FIGURE 2:**
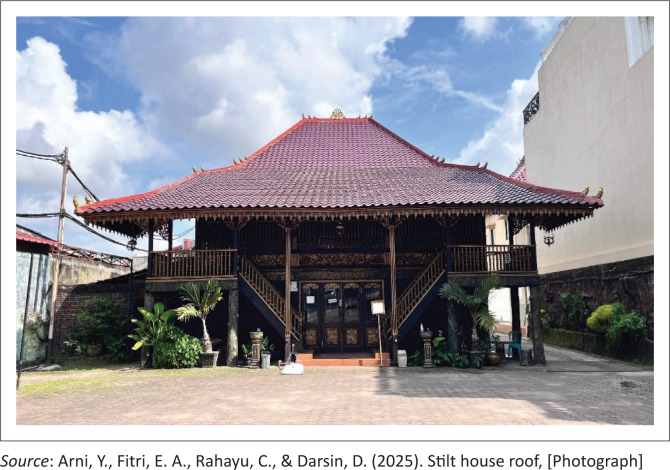
Stilt house roof.

In terms of roof structure, traditional houses often use a limp roof model or a saddle with a steep slope (Figure 2). This design is not just an aesthetic choice but also has a scientific function to accelerate the flow of rainwater so as to reduce the risk of seepage and overload on the roof frame. In the context of strong wind disaster mitigation, the tapered and aerodynamic shape of the roof helps to reduce wind pressure on the building surface. Steep sloping roofs reduce rainwater buildup, which often leads to collapse on heavy structures. The long overhang serves as a water runoff protector, reducing the erosion of the foundation soil. The philosophy that underlies all of these designs comes from the wisdom of the community that has coexisted for centuries with dynamic environmental conditions.

The maintenance of stilt houses is a critical factor in ensuring their durability against extreme weather and biological threats, such as termite infestations. Traditionally, local communities preserve the wooden structure by applying diesel fuel and coconut coir to the surface of the wood to protect it from pest damage. Additionally, the simple use of household detergents helps prevent the growth of moss, which can compromise both the aesthetics and structural integrity of the wood. Material-wise, routine treatment using solar and coconut fibre can extend the service life of the wood to more than 40 years. This finding is consistent with the study by Qian, Wang and Yu (2024), which revealed that the conservation of stilt houses in Hubei, China, depends largely on traditional maintenance practices that preserve the wood’s elastic modulus (Qian et al. 2024). Therefore, South Sumatran stilt houses are not only technically adaptive through features such as elevation, flexibility and earthquake load distribution but also ecologically sustainable and socially resilient. Irwanto et al. (2025) emphasise that caring for stilt houses is essential for ensuring their resilience against extreme weather and pest attacks. The use of diesel to preserve wood and detergent to prevent moss reflects consistent maintenance practices observed during field observations and interviews with local residents.

This statement underscores that regular care not only extends the lifespan of stilt houses but also reflects public awareness of the importance of maintaining sustainable, safe and comfortable living environments in the long term.

The traditional stilt house of the South Sumatran people has a distinctive structure and is full of philosophical meaning, especially related to the concept of *kekijing*, which is the value that regulates politeness and manners in social space. The division of space in this house is not only functional but also reflects the social hierarchy and cultural norms upheld by the community. In general, stilt houses consist of five levels of floors, each with its own symbolic function and meaning. This vertical load division has engineering implications. Where the lightest load is placed at the highest level, lowering the moment of inertia of the building during an earthquake. In addition, the most private room (Elephant Room) is at the most stable structural point, with a small number of openings similar to the concept *of an earthquake-proof house inner core*. Just like the high roof in the Joglo house provides space for evacuation, the multi-storey space in the stilt house provides a drop-safe zone area when the structure is deformed.

**FIGURE 3:**
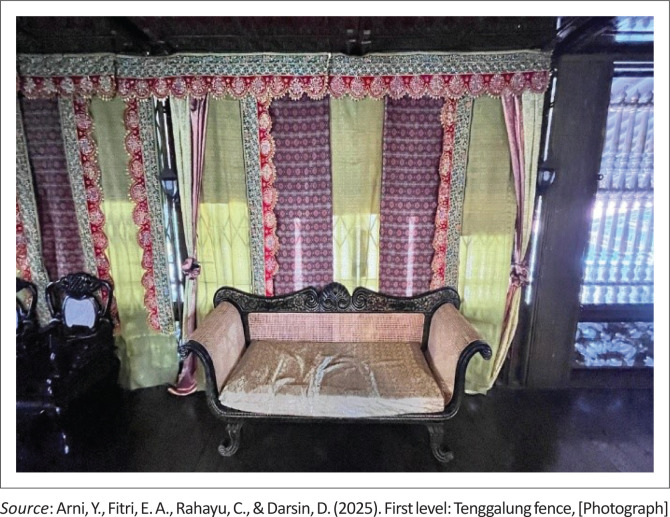
First level: *Tenggalung* fence.

The first level is known as the *Tenggalung* fence (Figure 3). This space functions as a welcoming area for guests and is open without wall boundaries, resembling a front porch or courtyard. The atmosphere at this level is typically informal and relaxed. It is often used to receive daily visitors or guests who arrive spontaneously. This wall-less space not only serves as a reception area but also allows for optimal airflow and light, reducing humidity that can trigger damage to the wood structure.

**FIGURE 4:**
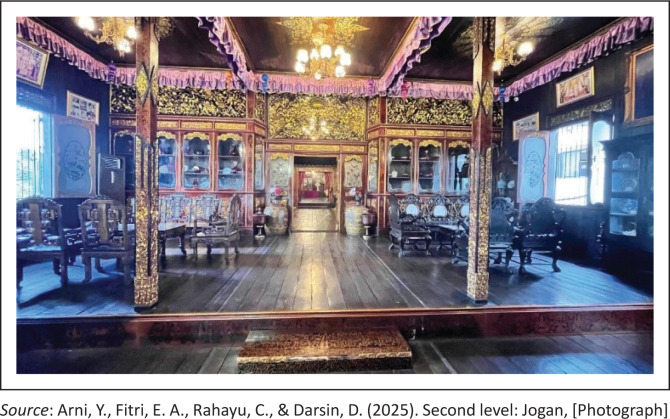
Second level: *Jogan*.

The second level is called *Jogan* (Figure 4), a space specifically designated for male family members. This area is more private in nature and serves as a spatial boundary that reflects the roles and responsibilities of men within the family structure. The division of rooms at this level demonstrates respect for gender identity and social roles within the community. In both personal and social contexts, the people of Palembang place great importance on honour and uphold strong customary norms that govern interactions between men and women.

**FIGURE 5:**
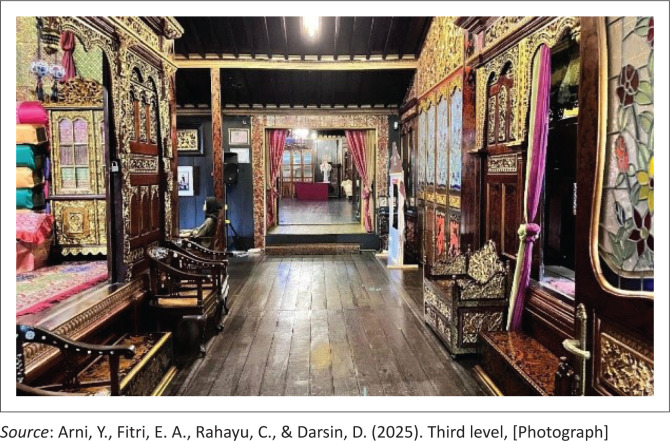
Third level.

The third level serves as a formal guest reception room, designated for special invitees (Figure 5). Positioned deeper within the house and elevated higher than the previous floor levels, this space is typically used during official events or family celebrations. It is reserved exclusively for guests with close kinship ties or specific social proximity, particularly elders or respected individuals. A partition wall is often used to maintain privacy and uphold the social order of the space.

**FIGURE 6:**
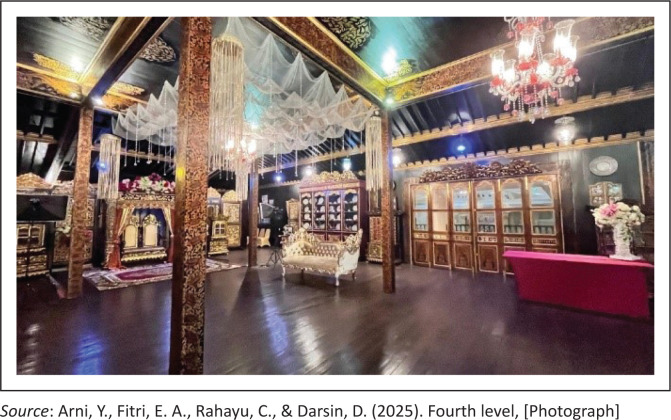
Fourth level.

The fourth level is reserved for close and respected relatives, particularly during traditional events such as communal feasts or ritual offerings [*selamatan* or *sedekah*] (Figure 6). This floor is elevated higher than the previous guest room, symbolising the honour and respect given to distinguished guests such as *Dapunto* [nobles] or elder family members (grandfather). Any inconsistency in guest placement at this level may lead to discomfort or even be perceived as a violation of established social norms.

**FIGURE 7:**
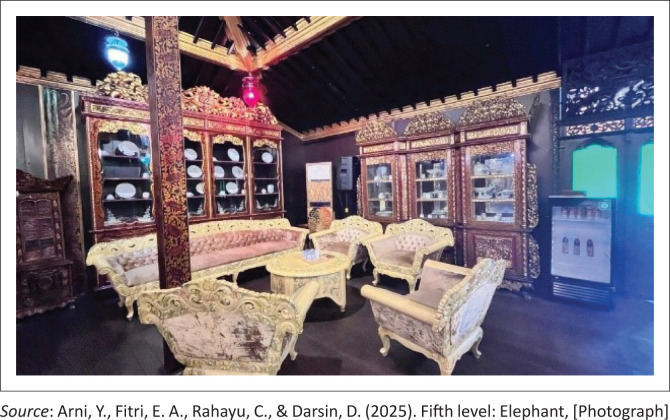
Fifth level: Elephant.

The fifth level, *known as the Elephant Room* [*Ruang Gajah*] (Figure 7), is the highest and most sacred space within the structure of a traditional stilt house. This room is exclusively reserved for community leaders, the head of the household or the nuclear family during important events such as major deliberations or wedding ceremonies.

The tiered structure of the traditional stilt house from the *Tenggalung* fence to the *Elephant Room* not only reflects the social hierarchy [*kekijing*] but also optimises mass distribution within the building layout. Placing lighter sections on the uppermost floors helps reduce moment inertia and torsion, resulting in a more stable response during earthquakes. Moreover, the tiered design of stilt houses is not merely an aspect of traditional architecture; it also represents the social order, value systems and cultural worldview of the South Sumatran people. As explained by Qian et al. (2024), traditional stilt house structures symbolically represent the resident’s social status and the deep interconnection between spatial organisation, customs and local cultural values (Qian et al. 2024). Thus, the stilt house functions not only as a dwelling but also as a tangible embodiment of intergenerational local wisdom.

### Social and cultural values

Stilt houses in South Sumatra reflect a profound philosophy of harmony between humans, nature and the Creator, often associated with the concept of *tri tangtu* or its local cultural equivalents. The spatial layout of the house also illustrates a clear social order: the front area is designated for receiving guests and a meeting room, the central room serves as a space for family gatherings and the rear area is reserved for domestic and spiritual activities. This spatial organisation represents a hierarchical yet harmonious model of social life. The concept of *kekijing* within the house structure carries deep philosophical meaning: beyond serving as a physical reinforcement of the building, *kekijing* embodies the principle of emotional and moral boundaries in human life. It symbolises the need for internal moral and social regulators, such as norms, values, and ethical boundaries, that guide human behavior and maintain social harmony. This philosophy aligns with the core social values of South Sumatran communities, which emphasise mutual cooperation [*gotong royong*], consensus [*musyawarah*] and a life guided by customary law [*adat*].

One important aspect of the philosophical foundation of traditional houses in South Sumatra is the orientation of the house. Many traditional stilt houses, particularly those along the banks of the Musi River, are constructed facing directly towards the river. This orientation is not solely based on practical considerations, such as easier access to water transportation or clean water sources, but also carries deep symbolic meaning. For the people of South Sumatra, the river is not merely a body of water; it represents life, sustenance, civilisation and social connectivity. Facing the river symbolises openness to life and a harmonious relationship with nature as the source of all power and vitality.

The strategic orientation of traditional houses also reflects the way local communities perceive space through a cosmological lens. The river is positioned as the central axis of life. Various traditional cultural rituals and activities, such as *Balimau* cleansing baths, offerings to the river and even funeral rites, are closely associated with the river’s direction. Thus, a house facing the river becomes a spiritual symbol of attachment to the natural cycle of life, from birth to death. This perspective aligns with the ethnoecological worldview that humans are an integral part of nature, not its dominators.

Equally important is the roof design of traditional South Sumatran houses, often shaped like a pyramid or a high, towering saddle. This architectural form carries deep philosophical meaning. The upward-reaching roof symbolises aspirations for prosperity, dignity and spiritual connectedness with God. In local tradition, the roof is seen as a symbol of the sky, while the house floor represents the earth. Thus, the house as a whole becomes a microcosmic representation of the universe a space where humans live between heaven and earth, in a state of balance and harmony.

### Level of youth knowledge on stilt houses

Based on a survey of 158 young people from local communities in the areas of Sumatra, it was found that their knowledge of stilt houses as a form of local wisdom and a disaster mitigation strategy is relatively high although certain aspects still require further strengthening. In general, the majority of the younger generation demonstrated a good understanding of the function of stilt houses in the context of flood and earthquake mitigation, as well as the social and cultural values embedded within them. However, in-depth understanding of the technical and symbolic aspects of stilt houses was not evenly distributed among all respondents. The results of the survey are presented in Table 1.

**TABLE 1:** The results of the survey.

Number	Question	Advance knowledge (% of respondents)	Adequate knowledge (% of respondents)	Minimum knowledge (% of respondents)	Do not have knowledge (% of respondents)
**Local community knowledge related to natural disasters**					
1	I know that the local community possesses knowledge about how to deal with floods.	29	69	2	0
2	I understand that traditional houses are designed to withstand natural disasters such as floods and earthquakes.	29	63	8	0
3	I know the natural signs commonly used by local communities to anticipate earthquakes or floods.	20	75	5	0
**Stilt house structure**					
4	I am aware of the natural signs commonly used by local communities to anticipate earthquakes or floods.	17	77	6	1
5	I understand that the structure of stilt houses is designed to reduce the risk of natural disasters such as floods and earthquakes.	35	58	7	0
6	I understand that the local materials used to build stilt houses can make them more resilient to natural disasters such as earthquakes and floods.	30	60	10	0
**Social and cultural values**					
7	I understand how the underfloor design of stilt houses functions during natural disasters.	22	71	5	1
8	I realise that stilt houses also serve as symbols of local cultural identity.	55	45	0	0
9	I know that stilt houses reflect the value of mutual cooperation in their construction.	46	53	0	0
**Economic function**					
10	I am aware of certain traditions or rituals associated with the construction of stilt houses.	24	61	15	0
11	I know that stilt houses have economic value because they use local materials.	36	62	3	0
**Stilt House Concept in Educational Context**					
12	I understand that the underfloor space of stilt houses can be used to store food reserves, rice supplies and other essential items.	28	59	11	3
13	I agree that stilt houses can be used as a learning medium for disaster mitigation.	31	67	2	1
14	Stilt houses can be integrated into science learning.	29	64	6	1

In terms of local community knowledge about disasters, 69% of respondents stated that they have adequate knowledge of how local people deal with floods, while 29% reported having advanced knowledge. The younger generation also has a fairly good understanding that traditional houses, such as stilt houses, are functionally designed to face disaster conditions like floods and earthquakes, with 63% indicating adequate knowledge and 29% indicating advanced knowledge. However, only 20% of respondents reported having a high level of advanced knowledge about natural signs commonly used by local communities to anticipate disasters. This indicates that ecological knowledge based on intergenerational experience is beginning to erode, as highlighted by Markolinda (2025), who emphasised the importance of passing down traditional knowledge to younger generations in disaster-prone areas (Markolinda 2025).

In terms of understanding the structure of stilt houses, the majority of the younger generation recognises their function as a form of disaster mitigation. As many as 35% of respondents reported having advanced knowledge about the function of stilt houses in reducing the impact of floods and earthquakes, while 58% had adequate knowledge. Knowledge about the local materials used in stilt house construction was also relatively high, with 30% of respondents demonstrating excellent knowledge and 60% sufficient knowledge. However, only 17% had an advanced understanding of how natural signs can be used as indicators of impending disasters. This is concerning, as according to Anwar (2025), stilt houses and knowledge of natural signs are integral parts of a traditional mitigation system proven to be adaptive to seismic and hydrometeorological risks in Sumatra (Anwar 2025).

In the social and cultural dimension, the awareness of the younger generation is relatively high. As many as 55% of respondents recognised that stilt houses are symbols of local cultural identity, and 46% associated stilt houses with the value of mutual cooperation in their construction. However, only 22% of respondents had an advanced understanding of the function of the underfloor space during disasters, such as its use for evacuation or logistics storage. This indicates that the symbolic and functional understanding of stilt houses in the context of disaster mitigation is still not sufficiently embedded in the younger generation, particularly among those living in urban or semi-urban areas where stilt houses are no longer the dominant architectural form.

From the economic and spiritual aspects, 36% of respondents understood that stilt houses have economic value because they utilise local materials, and 24% were aware of certain rituals or traditions involved in the construction process. This indicates a growing awareness of the sustainability and local values embedded in traditional buildings although not all respondents recognised the spiritual dimensions historically associated with the development of stilt houses. As noted by Basri (2021), the tradition of building houses is not only related to technical aspects but also to sacred values that strengthen the connection between humans and nature in the context of disaster resilience.

In the educational context, the younger generation shows strong potential in accepting the integration of stilt houses as a learning medium. As many as 67% of respondents had sufficient knowledge, and 31% had advanced knowledge in recognising stilt houses as a medium for disaster mitigation learning. Additionally, 64% agreed that stilt houses can be integrated into science education. This opens up opportunities for the development of ethnoscience-based curricula that link physical science concepts (such as force, pressure and structural balance) with local cultural realities. Research by Sandika (2025) supports the integration of local knowledge into learning as a strategy to strengthen disaster literacy among younger generations (Sandika 2025). Furthermore, analysis by age group showed that respondents aged 17–20 years had a higher level of structural knowledge than the age group of 21–25 years. This difference indicates that proximity to traditional home environments and direct experience have an effect on the retention of local knowledge. The same thing is found when comparing educational backgrounds; respondents with a high school or MA education background have a better understanding of the function of the house during a flood than students who live in urban areas.

Thus, the results show that the level of knowledge among the younger generation in South Sumatra regarding stilt houses falls within the moderate to high category. However, systematic efforts are still needed to enhance their understanding of the technical, symbolic and cosmological aspects of stilt houses, particularly through contextual educational approaches that connect local culture with modern science. Stilt houses, in this regard, are not merely cultural heritage but also represent adaptive solutions to the growing risks of disasters in coastal areas. These findings suggest that ethnoscience is derived primarily through social interaction and physical proximity, so that the farther a person is from the cultural environment, the lower his or her understanding of symbolic value.

Based on the perspective of resilience and adaptability capacity of the structural characteristics of stilt houses, it is proven to have a high adaptive capacity to floods and earthquakes through building elevation, wood flexibility and roof design that breaks down wind and rain pressure. Although respondents understood the practical function, the understanding that the design was the result of the accumulation of adaptive knowledge across generations was still relatively low. The survey results also showed that respondents who had been involved in family discussions about disasters had 12% – 18% higher levels of knowledge than those who had never discussed. This confirms that stilt houses function not only as physical structures but also as a medium for the transmission of values, norms and knowledge of disasters from generation to generation.

The findings of this study make it clear that the architecture of stilt houses not only reflects aesthetics or traditions but is a tangible form of adaptation of local communities to disaster-prone environments. Building elevations reduce flood exposure, flexible timber structures minimise earthquake damage and steep roof designs help drain rainwater efficiently. These three aspects show a strong relationship between traditional architectural practices, adaptive capacity and DRR strategies. Thus, knowledge about stilt houses can be one of the culturally relevant disaster education for the younger generation in the modern era.

This study is also aligned with the Sustainable Development Goals (SDGs), particularly SDG 4 (Quality Education), SDG 11 (Sustainable Cities and Communities) and SDG 13 (Climate Action), through the integration of local wisdom, specifically stilt house architecture into culturally based disaster mitigation education. In addition, this study supports the implementation of the Sendai Framework for Disaster Risk Reduction 2015–2030, especially its first and third priorities: enhancing disaster risk understanding and promoting locally-based investments for risk reduction by utilising traditional architecture as part of community resilience strategies against floods and earthquakes.

## Conclusion

The results of the study show that the architecture of stilt houses in South Sumatra is a form of effective technical adaptation to the danger of floods and earthquakes through structural elevation, flexibility of wood materials and the design of the basement as a water dam. In addition, stilt houses also represent cultural identity, as seen from the spatial orientation, building level layout and social functions that reflect the values and order of the local community. Based on the results of the questionnaire, it is emphasised that although the community understands the practical function of stilt houses, their understanding of the symbolic and philosophical aspects is still limited, thus opening up important opportunities for educational integration, namely incorporating this local knowledge into the DRR curriculum so that the younger generation is able to appreciate and utilise it as a mitigation strategy based on local wisdom.

Thus, the integration of stilt houses into science education based on ethnoscience can serve as a strategic solution to foster a culture of disaster preparedness and strengthen community resilience. The key recommendation from this study is the need to develop a local curriculum that links traditional architectural knowledge with disaster concepts, along with providing training for teachers and students to understand local values as part of disaster literacy. This aligns with the Sendai Framework and the School-Based Disaster Preparedness Programme (SSB) while also enhancing community resilience through educational pathways.
